# A survey of barriers and facilitators to the adoption of buprenorphine prescribing after implementation of a New Jersey-wide incentivized DATA-2000 waiver training program

**DOI:** 10.1186/s12913-024-10648-2

**Published:** 2024-02-08

**Authors:** Amesika N. Nyaku, Erin A. Zerbo, Clement Chen, Nicole Milano, Barbara Johnston, Randall Chadwick, Stephanie Marcello, Kaitlan Baston, Rachel Haroz, Stephen Crystal

**Affiliations:** 1 Department of Medicine, Division of Infectious Diseases, Rutgers New Jersey Medical School, 185 South Orange Ave, MSB I689, Newark, NJ 07103 USA; 2 Private Practice, Montclair, NJ 07042 USA; 3 Department of Psychiatry, Rutgers New Jersey Medical School, 183 South Orange Ave, BHSB F-Level, Newark, NJ 07103 USA; 4 Mental Health Association in New Jersey, 673 Morris Avenue, Suite 100, Springfield, NJ 07781 USA; 5 Rutgers University Behavioral Health Care, 151 Centennial Avenue, Suite 1140, Piscataway, NJ 08854 USA; 6 Department of Internal Medicine, Cooper Medical School of Rowan University, Three Cooper Plaza, Camden, NJ 08103 USA; 7 Department of Emergency Medicine, Cooper Medical School of Rowan University, One Cooper Plaza, Camden, NJ 08103 USA; 8 Center for Health Services Research, Institute for Health, Health Care Policy and Aging Research, Rutgers, the State University of New Jersey, 112 Paterson St, 3rd Floor, New Brunswick, NJ 08901 USA

**Keywords:** Survey, Buprenorphine, Opioid use disorder, Prescribing, Barriers, Facilitators, Office, Based addiction treatment

## Abstract

**Background:**

Opioid-involved overdose deaths continue to rise in the US, despite availability of highly effective treatments for opioid use disorder (OUD), in part due to the insufficient number of treatment providers. Barriers include the need for providers to gain expertise and confidence in providing MOUD to their patients who need these treatments. To mitigate this barrier, New Jersey sponsored a buprenorphine training program with financial incentives for participation, which met the then existing requirement for the DATA-2000 waiver. In a 2019 follow-up survey, participants reported on barriers and facilitators to subsequent buprenorphine prescribing.

**Methods:**

Participants in the training program completed a 10-min electronic survey distributed via email. The survey addressed demographics, practice characteristics, current buprenorphine prescribing, and barriers and facilitators to adoption and/or scale up of buprenorphine prescribing.

**Results:**

Of the 440 attendees with a valid email address, 91 individuals completed the survey for a response rate of 20.6%. Of the 91 respondents, 89 were eligible prescribers and included in the final analysis. Respondents were predominantly female (*n* = 55, 59.6%) and physicians (*n* = 55, 61.8%); representing a broad range of specialties and practice sites. 65 (73%) of respondents completed the training and DEA-registration, but only 31 (34.8%) were actively prescribing buprenorphine. The most frequently cited barriers to buprenorphine prescribing were lack of access to support services such as specialists in addiction, behavioral health services, and psychiatry. The most frequently reported potential facilitators were integrated systems with direct access to addiction specialists and psychosocial services, easier referral to behavioral health services, more institutional support, and improved guidance on clinical practice standards for OUD treatment.

**Conclusion:**

More than half (52.3%) of those who completed incentivized training and DEA registration failed to actively prescribe buprenorphine. Results highlight provider perceptions of inadequate availability of support for the complex needs of patients with OUD and suggest that broader adoption of buprenorphine prescribing will require scaling up support to clinicians, including increased availability of specialized addiction and mental health services.

**Supplementary Information:**

The online version contains supplementary material available at 10.1186/s12913-024-10648-2.

## Background

In the US, opioid-involved overdose deaths continue to precipitously increase each year with an increase from 47,600 in 2017 to 80,411 in 2021 [1–3]. Medications for opioid use disorder (MOUD) dramatically decrease opioid-involved overdoses [4, 5] and are a powerful tool to curb this public health crisis. However, despite the surging number of deaths, the proportion of individuals with an opioid use disorder (OUD) receiving MOUD remained stagnant at approximately 20% from 2016–2021 [6, 7]. The limited uptake of MOUD is partially related to limited treatment availability due to the additional regulations on the prescribing of methadone and buprenorphine for OUD. Until 2023, medical providers were required to obtain a Drug Addiction Treatment Act of 2000 (DATA-2000) waiver in order to prescribe buprenorphine-based OUD treatment in an office-based setting. This required that providers complete 8 to 24 h of training and then register with the Drug Enforcement Agency (DEA). The training and registration process for the DATA-2000 waiver has been documented as one barrier to adoption and expansion of OUD treatment [8–10], with less than 5% of eligible providers having DATA-2000 waivers [11] and many areas with high overdose rates having an inadequate supply of DATA-2000 waivered providers [11–13]. Some improvement in provider availability may emerge from the 2023 elimination of the DATA-2000 waiver training and requirement [14], however, these requirements have represented only a portion of the barriers to broader MOUD prescribing [15–17].

New Jersey has numbered amongst the states with the highest opioid overdose rates with 30 deaths per 100,000 of the population [18] and in an effort to increase the number of buprenorphine providers, New Jersey has implemented a series of interventions to decrease barriers to buprenorphine prescribing. This includes eliminating medication prior authorizations for buprenorphine for all Medicaid plans as has been done in other states including Illinois and California, and implementing higher reimbursement for office-based addiction treatment as in Virginia and Massachusetts [19, 20]. Financial incentives are often used to influence medical providers’ behaviors and a one-time incentive to obtain a DATA-2000 waiver has been piloted in Colorado, Pennsylvania and California [21–24]. In 2019, New Jersey provided financial incentives to medical providers who obtained their DATA-2000 waiver after completing a state facilitated waiver training course. The objective of this study is to evaluate barriers and facilitators to buprenorphine prescribing among individuals who participated in this incentivized waiver training program.

## Methods

### Study design

In 2019, New Jersey implemented an incentivized waiver training program that was funded by the Substance Abuse and Mental Health Services Administration (SAMHSA). Participants attended a 6-h in-person training which included 4 h of buprenorphine training and an additional 2-h information session about state-specific resources to support office-based addiction treatment providers. Participants who subsequently completed an additional 4-h online training and registered with the DEA received a $750 incentive. Trainings were open to a variety of medical professionals, though only individuals eligible to be buprenorphine prescribers were eligible for the incentive. This was a cross-sectional study of individuals that attended one of the 14 trainings held between May and November 2019.

### Study population and survey administration

All attendees who provided a valid email address at the time of training registration received a single, 10-min online survey distributed via REDCap [25] between February 2020 to July 2020. Non-respondents received an email reminder, approximately a week after initial distribution and a reminder phone call at their practice site, approximately 2 weeks later. Contact information for their clinical practice site was obtained from publicly available sources such as practice or university websites, online searches, and the SAMHSA data waiver directory. There was no compensation for participation.

### Survey development

We adapted the survey from prior surveys that assessed medical practitioners’ barriers, facilitators, and attitudes towards prescribing buprenorphine and caring for patients with OUD in a yes or no format [26, 27]. Additional questions were included regarding the respondent demographics and practice characteristics. Branching logic was used to capture the extent to which they completed the DATA-2000 waiver training and registration requirements, their current waiver limit (maximal allowable patients treated at one time of 30, 100, or 275), and buprenorphine prescribing (see Additional file 1).

### Statistical analysis

The participants’ demographic and practice characteristics, completion of the training and registration, and barriers and facilitators were first analyzed using descriptive statistics. Continuous data was analyzed using mean and standard deviation, except if not normally distributed; then median and interquartile range were used. Categorical data were described using frequency of counts. Because the group that completed the trainings but did not register with the DEA was less than 10 individuals, the 4 groups (in-person training only, completed both trainings but not registered, registered and not prescribing, and prescribing buprenorphine) were collapsed into dichotomous groups based on completion of training and DEA registration. Univariate logistic regression was done to assess for to determine if they had a statistically significant association with completion of the waiver training course and prescribing buprenorphine to patients. Results are reported as unadjusted odds ratios with associated 95% confidence interval. Statistical significance is defined as a *p*-value < 0.05. Statistical analyses were performed using Stata 17.

### IRB approval

The study was deemed exempt by the Rutgers Behavioral Health Sciences Institutional Review Board.

## Results

### Demographics and practice characteristics

**Fig. 1:**
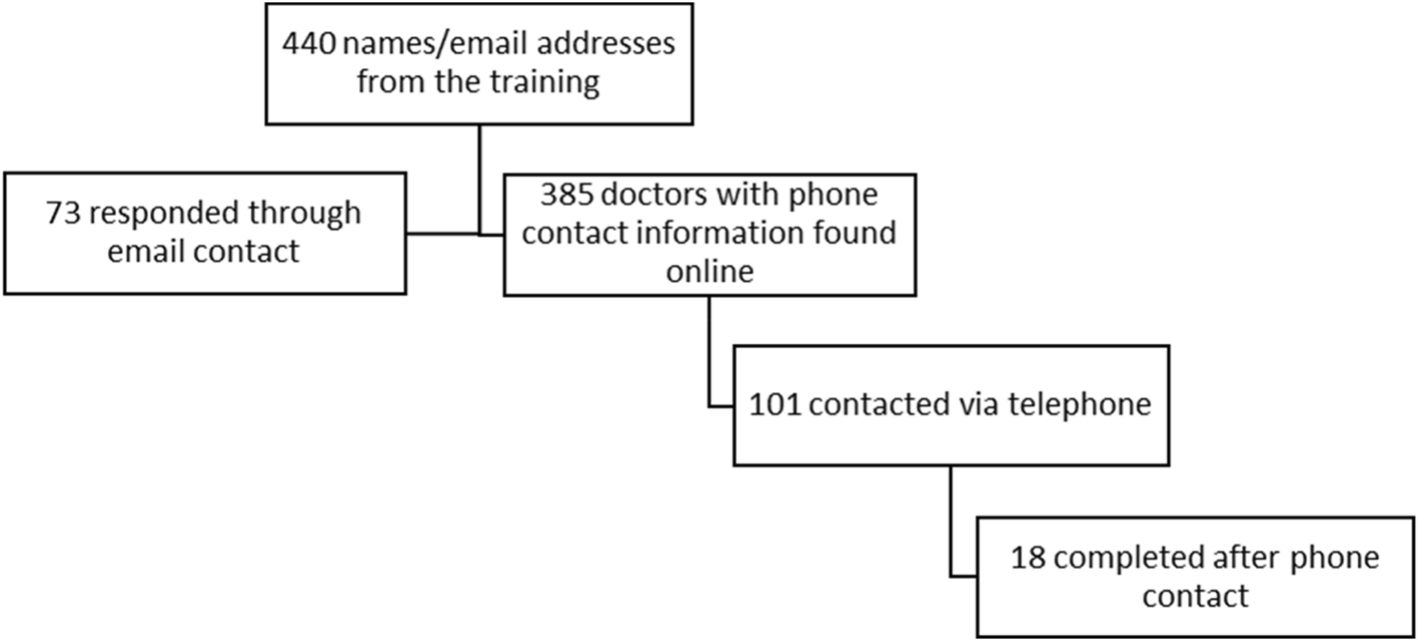
Flow diagram of survey respondents

Four hundred forty email addresses were collected, and 73 individuals completed the survey after the initial email contact or first reminder. Three hundred eighty-five individuals had publicly available contact information and 101 were successfully contacted via telephone which resulted in an additional 18 completed surveys (Fig. 1). There were a total of 91 respondents for a response rate of 20.6%. Of the 91 respondents, 89 were eligible to be buprenorphine prescribers and were included in the final analysis. Demographic information is reported in Table 1. Fifty-three (59.6%) respondents were female. Of the 83 participants that provided information about year of birth, the median year was 1974 (interquartile range [IQR] 1962–1983). Most respondents were physicians (*n* = 55, 61.8%), followed by 26 (29.2%) nurse practitioners, and 8 (9.0%) physician assistants. A diverse range of specialties were represented with 35 (39.3%) respondents from emergency medicine, 18 (20.2%) from family medicine, 15 (16.9%) from psychiatry, 14 (15.7%) from internal medicine, and the remainder from pediatrics, obstetrics, and pain management. The most common practice settings were the emergency department (*n* = 26, 29.2%), hospital (*n* = 24, 27.0%), and office-based group practice (*n* = 22, 24.7%). Self-reported information about payor source was provided by 82 respondents: nearly all accepted Medicaid (*n* = 75, 91.5%), Medicare (*n* = 80, 97.6%), and commercial insurance plans (*n* = 78, 95.1%) (Table 1).

**Table 1:** Respondent demographics and practice characteristics (*N* = 89)

Characteristic		
Year of birth^a^	1974 (IQR 1962–1983)	
Gender		
Male	36	40.5%
Female	53	59.6%
Profession		
Physician	55	61.8%
Nurse practitioner	26	29.2%
Physician assistant	8	9.0%
Specialty		
Internal Medicine	14	15.7%
Family Medicine	18	20.2%
Pediatrics	1	1.1%
Emergency Medicine	35	39.3%
Psychiatry	15	16.9%
Obstetrics and gynecology	1	1.1%
Pain management	4	4.5%
Other	1	1.1%
Practice setting		
Office-based solo practice	6	6.7%
Office-based group practice	22	24.7%
Licensed SUD treatment facility	2	2.3%
Hospital/healthcare system	24	27.0%
Emergency department	26	29.2%
Other	9	10.1%
Medicaid^b^		
Yes	75	91.5%
Medicare^c^		
Yes	80	97.6%
Private^d^		
Yes	78	95.1%
Cash^e^		
Yes	81	100.0%

*SUD* substance use disorder

Total respondents less than sample total: ^a^83, ^b^82, ^c^82, ^d^82, ^e^81

### Training, DEA-registration, and prescribing outcomes

Of the 89 respondents, 16 (18.0%) participated only in the in-person training, 8 (9.0%) completed both components of the training but did not register with the DEA, 34 (38.2%) completed the trainings and registration but were not prescribing buprenorphine, and 31 (34.8%) were actively prescribing buprenorphine. Of the 65 waivered respondents, 60 had a waiver limit of 30 patients and 5 had a waiver limit of 100 patients. Demographic or practice characteristics were not statistically significantly associated with whether respondents completed the waiver training or DEA registration.

### Barriers to adoption and expansion of buprenorphine prescribing

**Fig. 2:**
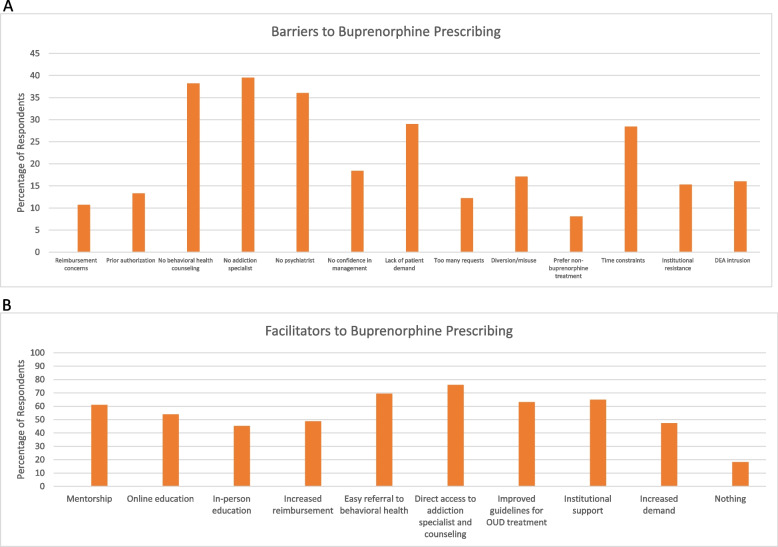
Barriers and facilitators to buprenorphine prescribing; **A** Barriers to buprenorphine prescribing. **B** Facilitators to buprenorphine prescribing

Out of the 89 respondents, 72 to 76 provided responses for the various questions about barriers to the adoption and expansion of buprenorphine prescribing. The most cited barriers to buprenorphine prescribing were lack of access to an addiction specialist (39.5%, *n* = 30), lack of access to behavioral health services (38.2%, *n* = 29), and lack of access to a psychiatrist (36%, *n* = 27). Other cited concerns were lack of patient demand, time constraints, lack of confidence in the management of patients with opioid use disorder, and concerns about buprenorphine misuse and/or diversion (Fig. 2A). Less frequently cited concerns included concern about DEA intrusion, resistance within their practice and/or lack of institutional support, prior authorization barriers, fear of too many treatment requests, low reimbursement, and preference for non-buprenorphine-based treatment options (Fig. 2A). For the individuals who did not complete the online training, 5 of 12 (41.7%) respondents reported technical problems that interfered with completing the online training. Table 2 shows the responses by group. Given the small number of respondents that completed only the in-person trainings or the trainings but did not register, the respondents were grouped according to whether they completed the training and registration. In the univariate analysis, individuals who completed the training and registered their DEA number were less likely to report concerns about DEA intrusion into their practice, OR = 0.21 (95% CI 0.06–0.78), *p* = 0.02 (Table 4). There were no other statistically significant differences between the groups.

**Table 2:** Barriers to buprenorphine prescribing by step completed in buprenorphine training, registration, and prescribing process

	Did not complete training	Did not register with DEA	Has DATA-2000, not prescribing buprenorphine	Prescribing buprenorphine
Reimbursement concerns (*n* = 75)	2 (18.2)	1 (16.7)	1 (3.6)	4 (13.3)
Prior authorization (*n* = 75)	1 (9.1)	2 (33.3)	3 (11.1)	4 (12.9)
No behavioral health counseling (*n* = 76)	4 (36.4)	1 (16.7)	9 (32.1)	15 (48.4)
No addiction specialist (*n* = 76)	6 (50)	3 (50)	8 (28.6)	13 (43.3)
No psychiatrist (*n* = 75)	5 (45.5)	2 (33.3)	6 (21.4)	14 (46.7)
No confidence in management (*n* = 76)	3 (27.3)	2 (33.3)	4 (13.8)	5 (16.7)
Lack of patient demand (*n* = 76)	3 (27.3)	1 (16.7)	14 (48.3)	4 (13.3)
Too many requests (*n* = 74)	1 (9.1)	0 (0)	6 (22.2)	2 (6.7)
Diversion/misuse (*n* = 76)	3 (25)	2 (33.3)	3 (10.7)	5 (16.7)
Prefer non-buprenorphine treatment (*n* = 74)	2 (18.2)	1 (16.7)	2 (7.4)	1 (3.3)
Time constraints (*n* = 74)	4 (36.4)	1 (16.7)	8 (29.6)	8 (26.7)
Institutional resistance (*n* = 72)	1 (10)	1 (16.7)	6 (22.2)	3 (10.3)
DEA intrusion (*n* = 75)	3 (27.3)	3 (50)	2 (7.1)	4 (13.3)

### Facilitators of adoption and expansion of buprenorphine prescribing

Out of the 89 respondents, between 73 and 78 provided responses for the questions about facilitators of the adoption and expansion of buprenorphine prescribing, with the exception that there were only 66 responses to the item “Nothing will increase my prescribing.” The most cited facilitators to buprenorphine prescribing were integrated systems with direct access to addiction specialists and psychosocial services (76%, *n* = 57), easier referral to behavioral health services (69.3%, *n* = 52), practice or institutional support for buprenorphine treatment (64.9%, *n* = 50), and improved guidance on clinical practice standards for treatment of opioid use disorder treatment (63%, *n* = 46) of respondents (Fig. 2B). Other desired supports included access to direct mentorship from an addiction specialist (61%, *n* = 47), additional online education (53.9%, *n* = 42), increased reimbursement (48.7%, *n* = 37), increased patient demand (47.4%, *n* = 36), and additional in-person training (45.3%, *n* = 34). A minority of individuals reported that nothing would increase their prescribing (18.2%,12 of 66) (Fig. 2B). When evaluating whether specific facilitators were associated with completing different stages of the waiver process and buprenorphine prescribing (i.e. in-person only training, completed both trainings but not registered, registered and not prescribing, and prescribing), respondents that completed the trainings but did not register or registered but were not prescribing were numerically more likely to report that there was nothing that would support their adoption and prescribing of buprenorphine (Table 3). However, in the univariate analysis, there were no facilitators that were significantly associated with completing the training and registration (Table 4).

**Table 3:** Facilitators to buprenorphine prescribing by step completed in buprenorphine training, registration, and prescribing process

	Did not complete training	Did not register with DEA	Has DATA-2000, not prescribing buprenorphine	Prescribing buprenorphine
Mentorship (*n* = 77)	8 (66.7)	4 (57.1)	17 (60.7)	18 (60)
Online education (*n* = 78)	11 (78.6)	4 (57.1)	11 (39.3)	16 (55.2)
In-person education (*n* = 75)	7 (63.6)	3 (50)	12 (42.9)	12 (40)
Increased reimbursement (*n* = 76)	7 (58.3)	3 (50)	14 (48.3)	13 (44.8)
Easy referral to behavioral health (*n* = 75)	7 (58.3)	3 (50)	20 (71.4)	22 (75.9)
Direct access to addiction specialist and counseling (*n* = 75)	9 (75)	4 (66.7)	19 (70.4)	25 (83.3)
Improved guidelines for OUD treatment (*n* = 73)	6 (54.6)	3 (50)	16 (59.3)	21 (72.4)
Institutional support (*n* = 77)	8 (66.7)	4 (57.1)	19 (67.9)	19 (63.3)
Increased demand (*n* = 76)	4 (36.4)	2 (33.3)	17 (58.6)	13 (43.3)
Nothing (*n* = 66)	0 (0)	3 (50)	7 (30.4)	2 (7.4)

**Table 4:** Unadjusted regression of barriers and facilitators to obtaining a DATA-2000 waiver

	OR		*p*	95% CI
Barriers				
Reimbursement concerns	0.44	0.3	0.09	2.07
Prior authorization	0.64	0.55	0.15	2.8
No behavioral health counseling	1.65	0.4	0.51	5.28
No addiction specialist	0.57	0.3	0.2	1.65
No psychiatrist	0.75	0.61	0.25	2.28
No confidence in management	0.43	0.19	0.12	1.53
Lack of patient demand	1.43	0.58	0.41	4.98
Too many requests	2.61	0.38	0.3	22.52
Diversion/misuse	0.42	0.18	0.12	1.49
Prefer non-buprenorphine treatment	0.26	0.12	0.05	1.43
Time constraints	0.94	0.91	0.28	3.09
Institutional resistance	1.34	0.73	0.26	6.94
DEA intrusion	0.21	0.02	0.06	0.78
Facilitators				
Mentorship	0.89	0.83	0.3	2.59
Online education	0.36	0.06	0.12	1.06
In-person education	0.49	0.21	0.16	1.48
Increased reimbursement	0.7	0.51	0.24	2.02
Easy referral to behavioral health	2.24	0.15	0.75	6.74
Direct access to addiction specialist and counseling	1.3	0.67	0.39	4.33
Improved guidelines for OUD treatment	1.73	0.33	0.58	5.21
Institutional support	1.11	0.85	0.38	3.26
Increased demand	1.89	0.26	0.62	5.80
Nothing	0.95	0.95	0.22	4.05

## Discussion

In this survey study, 89 of the 91 respondents were eligible for the DATA-2000 waiver, 73% completed the training and DEA registration requirements and 34.8% were prescribing buprenorphine after at least 4 months from their initial training. Though not encompassing all participants in the incentivized waiver program, only 48% of individuals who were trained and registered with the DEA were actively prescribing buprenorphine. This percentage does not differ from other estimates of actual buprenorphine prescribing among waivered providers (?) [15, 28–30]. In contrast to other studies which demonstrated differences by specialty and practice setting [10, 15, 26, 31], our study did not demonstrate any specific demographic and practice characteristics that were associated with completing the training and registration or initiating buprenorphine prescribing. Consistent with national trends, 39% of respondents were non-physician medical providers. Since the expansion of buprenorphine prescribing capabilities to non-physician medical providers in 2016 through the Comprehensive Addiction and Recovery Act, non-physician providers have increasingly accounted for a significant proportion of new providers seeking DATA-2000 waivers [32]. Additionally, a large proportion of respondents were emergency medicine providers. The large representation of emergency medicine providers may be related to these providers being exposed to opioid-related complications such as overdoses and serious bacterial infections and the increasing recognition that the emergency department can serve as valuable point of medical contact to help engage people with opioid use disorder in medical treatment [33–35].

Barriers to buprenorphine prescribing were similar across respondents irrespective of whether they completed the training, DEA registration, or were prescribing buprenorphine. The lack of access to addiction specialists, behavioral health services, and psychiatry were the most commonly identified barriers. These barriers are similar to those reported in prior studies across different specialty types, geographic locations, or populations served [36, 37]. Many of the policy interventions to address the opioid crisis have increased buprenorphine provider availability through the expansion of patient limits and eligible provider types, but the actualization of a sufficient specialty addiction and mental health workforce has not been realized [38–41]. The number of retiring psychiatrists, stagnant wages, dearth of culturally informed providers, and uneven geographic distribution of providers continue to be obstacles to the sufficient availability of specialty addiction and mental health providers [38, 40–42]. This workforce remains urgently needed to work in tandem with buprenorphine providers and to provide direct patient care. Further expansion of local and federal initiatives that support training and retention of this workforce are likely required [43, 44].

There was one barrier identified that was significantly associated with failure to obtain a DATA-2000 waiver: concerns about DEA intrusion into their practice. Respondents who did not complete the training and/or DEA registration were more likely to express concerns about DEA intrusion into their practice as a barrier. The legacy of enhanced scrutiny of buprenorphine prescribers [45] continues to impede efforts to scale up OUD treatment capacity [46]. Therefore, more national campaigns will be needed to reinforce the federal government’s support of widespread buprenorphine prescribing [47] or complete elimination of the DATA-waiver process [9].

Respondents identified similar potential facilitators for the adoption or expansion of buprenorphine prescribing. These facilitators primarily focused on system-level changes, including integrated systems with direct access to addiction specialists and psychosocial services, easier referral to behavioral health services, and increased practice/institutional support. Integration of physical and behavioral health can range from “screening and enhanced referral” to “care management with consultation” to “comprehensive treatment and population management” [48]. The level of integration is dependent on appropriateness for the local context based on the population needs and available physical, personnel, and financial resources [48]. The evidence has demonstrated the benefits of integration for people with opioid use disorder on patient related outcomes [49] and health system outcomes [50–52]. However, there is lack of widespread adoption. Local and federal financing that supports the implementation and sustainability of integrated care is needed. Financing strategies will require the removal of existing barriers such as prohibitions on same day billing for mental and physical services and limitations on the types of practice sites that are able to bill for mental or physical health services [53]. Additionally, incentives such has enhanced reimbursement [54] and value-based payment models [23] are needed to support the additional time that is inherently required to address complex co-occurring conditions. Our findings support the growing literature that indicates that providers are increasingly receptive to care integration and health system leaders need to accelerate their investment into these models [55, 56].

Respondents also indicated that individual level supports, such as more guidance on the treatment of OUD, direct mentorship by addiction specialists, and more online education opportunities were important facilitators to buprenorphine prescribing adoption and/or expansion. These findings confirm the findings of other surveys and qualitative interviews of DATA-2000 waivered providers that the DATA-2000 waiver training is not sufficient and that ongoing educational opportunities are necessary to help providers develop proficiency in the treatment of OUD [10, 15, 57].

There was a small subset of individuals that reported nothing would support their adoption and/or expansion of buprenorphine prescribing. Respondents who completed the training but were not registered or actively prescribing buprenorphine were more likely than other respondents to indicate that there was nothing that would support their adoption of buprenorphine prescribing. In Huhn and Dunn’s survey of waivered and non-waivered physicians, physicians who indicated that nothing would support their adoption of buprenorphine prescribing were more likely to also indicate concerns about being “inundated with requests? for buprenorphine” [10]. Saloner et al. proposed that office-based addiction treatment should be conceptualized into two tiers: providers who will widely expand their practice and providers who will only treat a few patients (mainly those already in their practice) [22]. It is possible that the ambivalence captured in our study represents the latter group and an alternative framework for medical provider engagement will be needed to engage this group in limited buprenorphine prescribing.

Our findings confirm the findings of other studies that the additional training required to be eligible for the DATA-2000 waiver continued to be an initial barrier to buprenorphine prescribing; 18% of respondents failed to complete all the educational components and, within that group, 41.7% reported technical difficulties as one barrier to course completion [10, 22, 58]. Though practice guidelines exist that allow qualifying providers to prescribe buprenorphine to no more than 30 patients at a single time without completing the DATA-2000 waiver training [59], the retention of the training requirement for providers to expand their patient panels may have had the unintended consequence of creating a new bottleneck which continues to impede widespread availability of buprenorphine [12, 60]. The passage of the Mainstreaming Addiction Treatment Act (MAT Act) is a much needed step towards increasing widespread access to addiction treatment [14]. However, our findings of barriers persisting even among providers who overcame the waiver barrier, and were sufficiently interested in MOUD to complete a formal training program, are indicative of the challenges that are likely to persist in broadening buprenorphine prescribing.

There are some limitations to our study and these findings should be cautiously interpreted due to the low response rate, which we hypothesize was in part related to the onset of the COVID-19 pandemic during the survey administration period. Additionally, there were variations in the response rates between the different groups, notably that respondents who did not complete the training or register with the DEA had lower response rates about their barriers to practice adoption, which may limit our understanding of the full range of barriers to practice adoption. Lastly, the survey did not assess how motivating the financial incentivize was for participation. Though the literature on the impact of financial incentives on DATA-2000 waiver training and subsequent buprenorphine prescribing have demonstrated that financial incentives increase training uptake, but results in minimal increases in actual buprenorphine prescribing without additional investments to support clinical care [22, 24].

## Conclusions

In this cross-sectional survey of participants who participated in the New Jersey sponsored and incentivized DATA-2000 waiver training program, nearly three-quarters of respondents completed the DATA-2000 training and DEA registration, but only 48% of DEA-registered respondents were actively prescribing buprenorphine. This rate of uptake is not significantly different from the findings from other studies of buprenorphine prescribing uptake. The system-level issues related to the lack of availability of comprehensive addiction and behavioral health treatment services and institutional buy-in are significant barriers. Results suggest that changes to these areas, such as enhanced availability of specialty addiction medicine and psychiatric referral with minimal wait time and immediate availability of counseling, social work and peer navigation services, may be needed for increased willingness to adopt and increase buprenorphine prescribing. Long-standing shortages of these services, exacerbated by increased needs in the wake of the COVID-19 pandemic, may require enhanced reimbursement and initiatives to improve supply and network adequacy for these providers, and increased integration of mental health services with MOUD provision. The rapid growth of tele-health modalities since the start of the pandemic could help to address geographic inequities in the distribution of providers. Results suggest that actualizing the potential of the MAT Act for broadened uptake of buprenorphine across healthcare settings, given the complex needs of the population with OUD, and provider perceptions of inadequate support, will call for robust efforts by healthcare payers, health plans and delivery systems to increase availability of clinical and care management support to providers of this greatly needed treatment.

### Supplementary Information

**Additional file 1.** Survey instruments.

## Data Availability

The datasets used and/or analyzed during the current study are available from the corresponding author on reasonable request.

## Abbreviations

OUD: Opioid use disorder
MOUD: Medications for opioid use disorder
IQR: Interquartile range
DEA: Drug Enforcement Agency
DATA-2000: Drug Addiction Treatment Act 2000
SAMHSA: Substance Abuse and Mental Health Services Administration
MAT Act: Mainstreaming Addiction Treatment Act of 2021
