# Supplementary material for: A survey of barriers and facilitators to the adoption of buprenorphine prescribing after implementation of a New Jersey-wide incentivized DATA-2000 waiver training program
Source: BMC Health Serv Res. 2024 Feb 8;24:179. doi: 10.1186/s12913-024-10648-2 (PMC10851589; doi:10.1186/s12913-024-10648-2)
Supplement: Supplementary file 1 — Additional file 1. Survey instruments. [file 12913_2024_10648_MOESM1_ESM.pdf]

# Additional file 1: Survey instruments

## Diagram of Survey Branching Logic

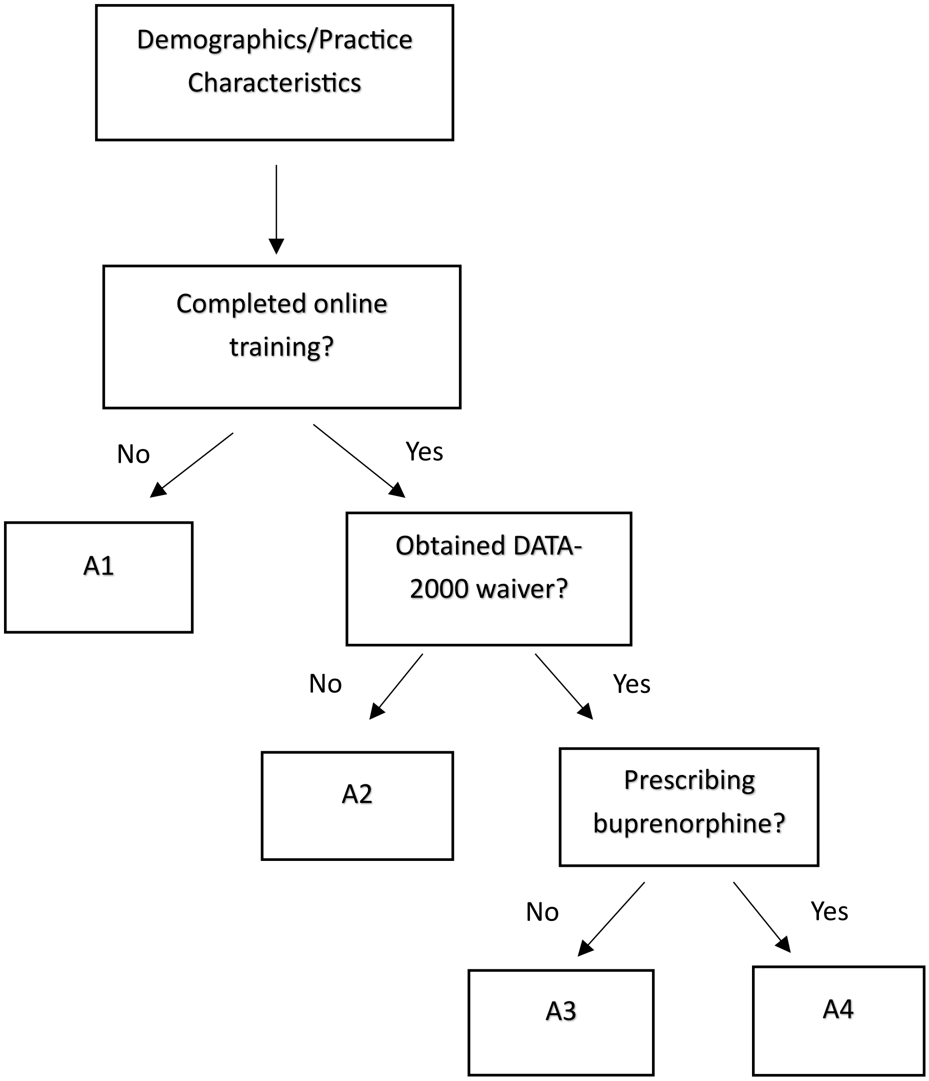


* indicates question has branching logic

# A1 – Survey instrument for respondents that did not complete training

| | |
|---|---|
| Record ID | __________________________________ |
| Please indicate the year you were born. | __________________________________ |
| Please indicate your gender. | ◯ Male<br>◯ Female<br>◯ Non-binary<br>◯ Prefer not to answer |
| What is the ZIP code of your principal practice? | __________________________________ |
| Please indicate your profession. | ◯ Physician<br>◯ Nurse Practitioner<br>◯ Physician Assistant<br>◯ Medical Trainee<br>◯ Other |
| What is your profession? | __________________________________ |
| Please indicate your medical specialty. | ◯ Internal Medicine<br>◯ Family Medicine<br>◯ Pediatrics<br>◯ Emergency Medicine<br>◯ Psychiatry<br>◯ Infectious Diseases<br>◯ Obstetrics and Gynecology<br>◯ Neurology<br>◯ Dermatology<br>◯ Pain Management<br>◯ Surgery<br>◯ Other |
| What is your medical specialty? | __________________________________ |
| Please indicate your principal practice setting. | ◯ Office-based solo practice<br>◯ Office-based group practice<br>◯ Licensed substance use disorder treatment facility<br>◯ Opioid Treatment Program (OTP)<br>◯ Hospital or healthcare system<br>◯ Publicly-funded clinic (FQHC, Rural Health Clinic, etc)<br>◯ Emergency Department<br>◯ Other |
| What is your practice setting? | __________________________________ |

**Please indicate the type(s) of insurance accepted at your practice.**

| | Yes | No |
|---|---|---|
| Medicaid | ◯ | ◯ |
| Medicare | ◯ | ◯ |
| Private insurance | ◯ | ◯ |
| Self pay | ◯ | ◯ |

Did you complete the online portion of the Provider's Clinical Support System for Medication-Assisted Treatment (PCSS-MAT) waiver training?

◯ Yes
◯ No

*

**Were any of the following issues a barrier to complete the training and register for your DATA 2000 waiver?**

| | Yes | No |
|---|---|---|
| Technical difficulties with the online waiver training | ◯ | ◯ |
| Reimbursement concerns | ◯ | ◯ |
| Prior authorization concerns | ◯ | ◯ |
| Lack of access to behavioral health services | ◯ | ◯ |
| Lack of access to addiction specialists | ◯ | ◯ |
| Lack of access to a psychiatrist | ◯ | ◯ |
| Lack of confidence in management of patients with opioid use disorder | ◯ | ◯ |
| Lack of patient demand | ◯ | ◯ |
| Concern about too many requests for buprenorphine | ◯ | ◯ |
| Concerned about risk of misuse or diversion of buprenorphine | ◯ | ◯ |
| Prefer non-buprenorphine treatment options | ◯ | ◯ |
| Time constraints in my practice | ◯ | ◯ |
| Resistance from practice partners/staff or lack of institutional support | ◯ | ◯ |
| Concerns over DEA intrusion into your practice | ◯ | ◯ |

## Would any of these incentives enable you to prescribe (more) buprenorphine?

| | Yes | No |
|---|---|---|
| Addiction specialist mentor | ◯ | ◯ |
| Additional online education on addiction treatment | ◯ | ◯ |
| Additional in-person education on addiction treatment | ◯ | ◯ |
| Increased reimbursement | ◯ | ◯ |
| Easier system for referral to behavioral health services | ◯ | ◯ |
| Integrated system with direct access to addiction specialists and psychosocial services | ◯ | ◯ |
| Improved guidance on clinical practice standards for treatment of opioid use disorder | ◯ | ◯ |
| Practice or institutional support for buprenophine treatment | ◯ | ◯ |
| Increased patient demand | ◯ | ◯ |
| Nothing will increase my prescribing | ◯ | ◯ |

# A2 – Survey instrument for respondents that completed training but did not complete DEA registration

Record ID

__________________________________

Please indicate the year you were born.

__________________________________

Please indicate your gender.

- ◯ Male
- ◯ Female
- ◯ Non-binary
- ◯ Prefer not to answer

What is the ZIP code of your principal practice?

__________________________________

Please indicate your profession.

- ◯ Physician
- ◯ Nurse Practitioner
- ◯ Physician Assistant
- ◯ Medical Trainee
- ◯ Other

What is your profession?

__________________________________

Please indicate your medical specialty.

- ◯ Internal Medicine
- ◯ Family Medicine
- ◯ Pediatrics
- ◯ Emergency Medicine
- ◯ Psychiatry
- ◯ Infectious Diseases
- ◯ Obstetrics and Gynecology
- ◯ Neurology
- ◯ Dermatology
- ◯ Pain Management
- ◯ Surgery
- ◯ Other

What is your medical specialty?

__________________________________

Please indicate your principal practice setting.

- ◯ Office-based solo practice
- ◯ Office-based group practice
- ◯ Licensed substance use disorder treatment facility
- ◯ Opioid Treatment Program (OTP)
- ◯ Hospital or healthcare system
- ◯ Publicly-funded clinic (FQHC, Rural Health Clinic, etc)
- ◯ Emergency Department
- ◯ Other

What is your practice setting?

__________________________________

**Please indicate the type(s) of insurance accepted at your practice.**

| | Yes | No |
|---|---|---|
| Medicaid | ◯ | ◯ |
| Medicare | ◯ | ◯ |
| Private insurance | ◯ | ◯ |
| Self pay | ◯ | ◯ |

Did you complete the online portion of the Provider's Clinical Support System for Medication-Assisted Treatment (PCSS-MAT) waiver training? ◯ Yes ◯ No *

Did you obtain your DATA 2000 waiver? ◯ Yes ◯ No *

**Are any of the following issues a barrier to registering for your waiver and prescribing buprenorphine?**

| | Yes | No |
|---|---|---|
| Reimbursement concerns | ◯ | ◯ |
| Prior authorization | ◯ | ◯ |
| Lack of access to behavioral health services | ◯ | ◯ |
| Lack of access to addiction specialists | ◯ | ◯ |
| Lack of access to a psychiatrist | ◯ | ◯ |
| Lack of confidence in management patients with opioid use disorder | ◯ | ◯ |
| Lack of patient demand | ◯ | ◯ |
| Did not want too many requests for buprenorphine | ◯ | ◯ |
| Concerned about risk of misuse or diversion of buprenorphine | ◯ | ◯ |
| Prefer non-buprenorphine treatment options | ◯ | ◯ |
| Time constraints in my practice | ◯ | ◯ |
| Resistance from practice partners/staff or lack of institutional support | ◯ | ◯ |
| Concerns over DEA intrusion into your practice | ◯ | ◯ |

**Would any of these incentives enable you to register for your waiver and prescribe buprenorphine?**

| | Yes | No |
|---|---|---|
| Addiction specialist mentor | ◯ | ◯ |
| Additional online education on addiction treatment | ◯ | ◯ |
| Additional in-person education on addiction treatment | ◯ | ◯ |
| Increase reimbursement | ◯ | ◯ |
| Easier system for referral to psychosocial services | ◯ | ◯ |
| Integrated system with direct access to addiction specialists and psychosocial services | ◯ | ◯ |
| Improved guidance on clinical practice standards for treatment of opioid use disorder | ◯ | ◯ |
| Practice or institutional support for buprenophine treatment | ◯ | ◯ |
| Increased patient demand | ◯ | ◯ |
| Nothing will increase my prescribing | ◯ | ◯ |

# A3 – Survey instrument for respondents that have an active DATA-2000 waiver but are not currently prescribing buprenorphine-based treatment

Record ID

__________________________________

Please indicate the year you were born.

__________________________________

Please indicate your gender.

- ◯ Male
- ◯ Female
- ◯ Non-binary
- ◯ Prefer not to answer

What is the ZIP code of your principal practice?

__________________________________

Please indicate your profession.

- ◯ Physician
- ◯ Nurse Practitioner
- ◯ Physician Assistant
- ◯ Medical Trainee
- ◯ Other

What is your profession?

__________________________________

Please indicate your medical specialty.

- ◯ Internal Medicine
- ◯ Family Medicine
- ◯ Pediatrics
- ◯ Emergency Medicine
- ◯ Psychiatry
- ◯ Infectious Diseases
- ◯ Obstetrics and Gynecology
- ◯ Neurology
- ◯ Dermatology
- ◯ Pain Management
- ◯ Surgery
- ◯ Other

What is your medical specialty?

__________________________________

Please indicate your principal practice setting.

- ◯ Office-based solo practice
- ◯ Office-based group practice
- ◯ Licensed substance use disorder treatment facility
- ◯ Opioid Treatment Program (OTP)
- ◯ Hospital or healthcare system
- ◯ Publicly-funded clinic (FQHC, Rural Health Clinic, etc)
- ◯ Emergency Department
- ◯ Other

What is your practice setting?

__________________________________

## Please indicate the type(s) of insurance accepted at your practice.

| | Yes | No |
|---|---|---|
| Medicaid | ◯ | ◯ |
| Medicare | ◯ | ◯ |
| Private insurance | ◯ | ◯ |
| Self pay | ◯ | ◯ |

Did you complete the online portion of the Provider's Clinical Support System for Medication-Assisted Treatment (PCSS-MAT) waiver training? ◯ Yes ◯ No *

Did you obtain your DATA 2000 waiver? ◯ Yes ◯ No *

Did you grant permission to be listed on the SAMHSA's Buprenorphine Provider Locator? ◯ Yes ◯ No

Please indicate your current DATA 2000 patient limit. ◯ 30 ◯ 100 ◯ 275

Have you prescribed buprenorphine for the treatment of opioid use disorder since obtaining a DATA 2000 waiver? ◯ Yes ◯ No *

## Are any of the following issues a barrier to prescribing buprenorphine?

| | Yes | No |
|---|---|---|
| Reimbursement concerns | ◯ | ◯ |
| Prior authorization | ◯ | ◯ |
| Lack of access to behavioral health services | ◯ | ◯ |
| Lack of access to addiction specialists | ◯ | ◯ |
| Lack of access to a psychiatrist | ◯ | ◯ |
| Lack of confidence in management patients with opioid use disorder | ◯ | ◯ |
| Lack of patient demand | ◯ | ◯ |
| Did not want too many requests for buprenorphine | ◯ | ◯ |
| Concerned about risk of misuse or diversion of buprenorphine | ◯ | ◯ |
| Prefer non-buprenorphine treatment options | ◯ | ◯ |
| Time constraints in my practice | ◯ | ◯ |

| | | |
|---|---|---|
| Resistance from practice partners/staff or lack of institutional support | ◯ | ◯ |
| Concerns over DEA intrusion into your practice | ◯ | ◯ |

**Would any of these incentives enable you to prescribe buprenorphine?**

| | Yes | No |
|---|---|---|
| Addiction specialist mentor | ◯ | ◯ |
| Additional online education on addiction treatment | ◯ | ◯ |
| Additional in-person education on addiction treatment | ◯ | ◯ |
| Increase reimbursement | ◯ | ◯ |
| Easier system for referral to psychosocial services | ◯ | ◯ |
| Integrated system with direct access to addiction specialists and psychosocial services | ◯ | ◯ |
| Improved guidance on clinical practice standards for treatment of opioid use disorder | ◯ | ◯ |
| Practice or institutional support for buprenophine treatment | ◯ | ◯ |
| Increased patient demand | ◯ | ◯ |
| Nothing will increase my prescribing | ◯ | ◯ |

# A4 – Survey instrument for respondents that have an active DATA-2000 waiver and are prescribing buprenorphine-based treatment

Record ID ______________________________

Please indicate the year you were born. ______________________________

Please indicate your gender.
- ◯ Male
- ◯ Female
- ◯ Non-binary
- ◯ Prefer not to answer

What is the ZIP code of your principal practice? ______________________________

Please indicate your profession.
- ◯ Physician
- ◯ Nurse Practitioner
- ◯ Physician Assistant
- ◯ Medical Trainee
- ◯ Other

What is your profession? ______________________________

Please indicate your medical specialty.
- ◯ Internal Medicine
- ◯ Family Medicine
- ◯ Pediatrics
- ◯ Emergency Medicine
- ◯ Psychiatry
- ◯ Infectious Diseases
- ◯ Obstetrics and Gynecology
- ◯ Neurology
- ◯ Dermatology
- ◯ Pain Management
- ◯ Surgery
- ◯ Other

What is your medical specialty? ______________________________

Please indicate your principal practice setting.
- ◯ Office-based solo practice
- ◯ Office-based group practice
- ◯ Licensed substance use disorder treatment facility
- ◯ Opioid Treatment Program (OTP)
- ◯ Hospital or healthcare system
- ◯ Publicly-funded clinic (FQHC, Rural Health Clinic, etc)
- ◯ Emergency Department
- ◯ Other

What is your practice setting? ______________________________

## Please indicate the type(s) of insurance accepted at your practice.

| | Yes | No |
|---|---|---|
| Medicaid | ◯ | ◯ |
| Medicare | ◯ | ◯ |
| Private insurance | ◯ | ◯ |
| Self pay | ◯ | ◯ |

Did you complete the online portion of the Provider's Clinical Support System for Medication-Assisted Treatment (PCSS-MAT) waiver training?
◯ Yes
◯ No
*

Did you obtain your DATA 2000 waiver?
◯ Yes
◯ No
*

Did you grant permission to be listed on the SAMHSA's Buprenorphine Provider Locator?
◯ Yes
◯ No

Please indicate your current DATA 2000 patient limit.
◯ 30
◯ 100
◯ 275

Have you prescribed buprenorphine for the treatment of opioid use disorder since obtaining a DATA 2000 waiver?
◯ Yes
◯ No
*

## Please indicate which of the following groups of patients you accept for buprenorphine treatment.

| | Yes | No | Not applicable |
|---|---|---|---|
| Patients who are already on your personal panel | ◯ | ◯ | ◯ |
| Patients of other clinicians in your clinic | ◯ | ◯ | ◯ |
| Patients new to your clinic | ◯ | ◯ | ◯ |

## Are any of the following issues a barrier to prescribing buprenorphine?

| | Yes | No |
|---|---|---|
| Reimbursement concerns | ◯ | ◯ |
| Prior authorization | ◯ | ◯ |
| Lack of access to behavioral health services | ◯ | ◯ |
| Lack of access to addiction specialists | ◯ | ◯ |
| Lack of access to a psychiatrist | ◯ | ◯ |
| Lack of confidence in management patients with opioid use disorder | ◯ | ◯ |

| | | |
|---|---|---|
| Lack of patient demand | ○ | ○ |
| Did not want too many requests for buprenorphine | ○ | ○ |
| Concerned about risk of misuse or diversion of buprenorphine | ○ | ○ |
| Prefer non-buprenorphine treatment options | ○ | ○ |
| Time constraints in my practice | ○ | ○ |
| Resistance from practice partners/staff or lack of institutional support | ○ | ○ |
| Concerns over DEA intrusion into your practice | ○ | ○ |

**Would any of these incentives enable you to prescribe buprenorphine to more patients?**

| | Yes | No |
|---|---|---|
| Addiction specialist mentor | ○ | ○ |
| Additional online education on addiction treatment | ○ | ○ |
| Additional in-person education on addiction treatment | ○ | ○ |
| Increase reimbursement | ○ | ○ |
| Easier system for referral to psychosocial services | ○ | ○ |
| Integrated system with direct access to addiction specialists and psychosocial services | ○ | ○ |
| Improved guidance on clinical practice standards for treatment of opioid use disorder | ○ | ○ |
| Practice or institutional support for buprenophine treatment | ○ | ○ |
| Increased patient demand | ○ | ○ |
| Nothing will increase my prescribing | ○ | ○ |
